# Validation List no. 227: valid publication of new names and new combinations effectively published outside the IJSEM

**DOI:** 10.1099/ijsem.0.006989

**Published:** 2026-01-30

**Authors:** Aharon Oren, Markus Göker

**Affiliations:** 1 The Institute of Life Sciences, The Hebrew University of Jerusalem, The Edmond J. Safra Campus, 9190401 Jerusalem, Israel; 2 Leibniz Institute DSMZ – German Collection of Microorganisms and Cell Cultures, Inhoffenstrasse 7B, 38124 Braunschweig, Germany

The purpose of this list is to publish in the *International Journal of Systematic and Evolutionary Microbiology* (*IJSEM*) those new names and new combinations that were effectively published outside the *IJSEM* as set forth in Rule 27 of the International Code of Nomenclature of Prokaryotes (2022 Revision) [1]. Authors and other individuals wishing to have new names and/or combinations included in future lists should send an electronic copy of the published paper to the IJSEM Editorial Office for confirmation that all of the other requirements for a valid publication have been met. It is also a requirement of *IJSEM* and the International Code of Nomenclature of Prokaryotes (ICNP) that authors of new species, new subspecies and new combinations provide evidence that types are deposited in two publicly accessible culture collections in two different countries. It should be noted that the date of valid publication of these new names and combinations is the date of publication of this list, not the date of the original publication of the names and combinations. The authors of the new names and combinations are as given below.

Inclusion of a name on these lists validates the publication of the name and thereby makes it available in the nomenclature of prokaryotes. Inclusion of a name on these lists is part of the requirements of valid publication of an effectively published name. Indeed, some of these names may, in time, be considered later synonyms, or it may be proposed to transfer the organisms to another genus, thus necessitating the creation of a new combination. Conversely, it may be proposed to transfer an organism back to an earlier genus and to regard the basonym or an earlier new combination as the correct name instead of a more recent new combination. The ICNP does not decide on such taxonomic opinions.

**Table:** 

Name/authors	Proposed as	Nomenclatural type^1^	Priority^2^	Reference
*Actinokineospora xionganensis* Wang *et al*. 2021, 493	sp. nov.	HBU206404 (=KCTC 49404=MCCC 1K04412)	25	[2]
*Actinospongiicola* corrig. Huang *et al*. 2025, 15^3,4^	gen. nov.	*Actinospongiicola halochondriae* corrig^5^.	59	[3]
*Actinospongiicola halichondriae* corrig. Huang *et al*. 2025, 15^3^	sp. nov.	Hal317 (=DSM 114536=LMG 32795)	59	[3]
*Aeromicrobium halotolerans* Yan *et al*. 2016, 426	sp. nov.	YIM Y47 (=DSM 29939=JCM 30627)^6^	34	[4]
*Affinundibacterium* Ma *et al*. 2023, 8^3^	gen. nov.	*Affinundibacterium seohonense*	99	[5]
*Affinundibacterium amnicola* (Chen *et al*. 2017) Ma *et al*. 2023, 9^3^	comb. nov. [basonym: *Undibacterium amnicola* Chen *et al*. 2017]	KYPY9 (=BCRC 81009=KCTC 52442)^7^	99	[5]
*Affinundibacterium baiyunense* (Lu *et al*. 2021) Ma *et al*. 2023, 9^3^	comb. nov. [basonym: *Undibacterium baiyunense* Lu *et al*. 2021]	BYS107W (=GDMCC 1.2453=KCTC 82653)	99	[5]
*Affinundibacterium cyanobacteriorum* (Le *et al*. 2025) Lu *et al*. 2025, 11^3^	comb. nov. [basonym: *Undibacterium cyanobacteriorum* Le *et al*. 2025]	20NA775 (=KCTC 8005=LMG 33136)	101	[6]
*Affinundibacterium danionis* (Kämpfer *et al*. 2016) Lu *et al*. 2025, 11^3^	comb. nov. [basonym: *Undibacterium danionis* Kämpfer *et al*. 2016]	E3/4 (=CCM 8677=CIP 111017)^8^	101	[6]
*Affinundibacterium fentianense* (Lu *et al*. 2021) Ma *et al*. 2023, 9^3^	comb. nov. [basonym: *Undibacterium fentianense* Lu *et al*. 2021]	FT137W (=GDMCC 1.2456=KCTC 82656)	99	[5]
*Affinundibacterium flavidum* (Lu *et al*. 2021) Ma *et al*. 2023, 9^3^	comb. nov. [basonym: *Undibacterium flavidum* Lu *et al*. 2021]	LX15W (=GDMCC 1.1910=JCM 34286)	99	[5]
*Affinundibacterium macrobrachii* (Sheu *et al*. 2014) Ma *et al*. 2023, 9^3^	comb. nov. [basonym: *Undibacterium macrobrachii* Sheu *et al*. 2014]^9^	CMJ-9 (=DSM 29770=KCTC 23916)^10^	99	[5]
*Affinundibacterium nitidum* (Lu *et al*. 2021) Ma *et al*. 2023, 9^3^	comb. nov. [basonym: *Undibacterium nitidum* Lu *et al*. 2021]	LX22W (=GDMCC 1.1912=KACC 21957)	99	[5]
*Affinundibacterium seohonense* (Kim *et al*. 2014) Ma *et al*. 2023, 9^3^	comb. nov. [basonym: *Undibacterium seohonense* Kim *et al*. 2014]	SHS5-24 (=KACC 16656=NBRC 108929)	99	[5]
*Agrobacterium deltae* corrig. Yan *et al*. 2017, 1007^11^	sp. nov.	YIC4121 (=HAMBI 3654=LMG 29283)	21	[7]
*Agromyces silvae* Lee *et al*. 2024, 1480	sp. nov.	W11 (=KCTC 49818=NBRC 115999)	108	[8]
*Algibacter ulvanivorans* Li *et al*. 2025, 10^3^	sp. nov.	PT7-4 (=KCTC 102077=MCCC 1K08669)	18	[9]
*Algoriphagus limi* Ahn *et al*. 2023, 4^3,12^	sp. nov.	CAU 1643 (=KCTC 92080=MCCC 1K07150)	91	[10]
*Alistipes senegalensis* Mishra *et al*. 2012, 313^13^	sp. nov.	JC50 (=CSUR P156=DSM 25460)	126	[11]
*Alteromonas aquimaris* Wang *et al*. 2023, 6^3,14^	sp. nov.	ASW11-7 (=KCTC 92853=MCCC 1K07240)	62	[12]
*Alteromonas nitratireducens* Chang *et al*. 2025, 16^3^	sp. nov.	CYL-A6 (=KCTC 8709=MCCC 1K09369)	61	[13]
*Alteromonas salexigens* Sun *et al*. 2023, 5^3^	sp. nov.	ASW 11–19 (=KCTC 92247=MCCC 1K07239)	142	[14]
*Anaerococcus vaginimassiliensis* Bordigoni *et al*. 2020, 6^3,15^	sp. nov.	Marseille-P4512 (=CCUG 71849=CSUR P4512)^16^	127	[15]
*Andreesenella* Poehlein *et al*. 2025, 1^3^	gen. nov.	*Andreesenella formicoacetica*	67	[16]
*Andreesenella acetica* (Gottschalk and Braun 1981) Poehlein *et al*. 2025, 1^3^	comb. nov. [basonym: *Clostridium aceticum* (*ex* Wieringa 1940) Gottschalk and Braun 1981]	DSM 1496 (=ATCC 35044)	67	[16]
*Andreesenella formicacetica* corrig. (Andreesen *et al*. 1970) Poehlein *et al*. 2025, 1^3,17^	comb. nov. [basonym: *Clostridium formicaceticum* corrig. Andreesen *et al*. 1970 (Approved Lists 1980)]	A1 (=ATCC 27076=DSM 92)	67	[16]
*Aquimarina besae* Kim *et al*. 2025, 10^3^	sp. nov.	SS2-1 (=DSM 119931=KACC 23859)	78	[17]
*Aquimarina rhodophyticola* Kim *et al*. 2025, 8^3^	sp. nov.	W85 (=DSM 119937=KACC 23858)	78	[17]
*Arvimicrobium* Lee *et al*. 2025, 6^3^	gen. nov.	*Arvimicrobium flavum*	93	[18]
*Arvimicrobium flavum* Lee *et al*. 2025, 6^3^	sp. nov.	1W2 (=KCTC 92441=NBRC 116019)	93	[18]
*Astrobacterium* Potokina *et al*. 2025, 7^3^	gen. nov.	*Astrobacterium formosum*	82	[19]
*Astrobacterium formosum* Potokina *et al*. 2025, 7^3^	sp. nov.	Astr-EGSB (=CGMCC 1.62199=VKM B-3749)	82	[19]
*Aurantiacibacter poecillastricola* Kim *et al*. 2025, 6^3,18^	sp. nov.	219JJ12-13 (=KACC 23236=LMG 33060)	109	[20]
*Aurantibrevibacter* Cao *et al*. 2025, 7^3,19^	gen. nov.	*Aurantibrevibacter litoralis*	16	[21]
*Aurantibrevibacter litoralis* Cao *et al*. 2025, 8^3^	sp. nov.	GZD-96 (=KCTC 102227=MCCC 1K08935)	16	[21]
*Bacillus nitroreducens* Guo *et al*. 2016, 351	sp. nov.	GSS08 (=KCTC 33699=MCCC 1K01091)	88	[22]
*Borrelia* (Swellengrebel 1907) Margos *et al*. 2025, 3^3^	subgen. nov.	*Borrelia anserina*	11	[23]
*Borreliella* (Adeolu and Gupta 2015) Margos *et al*. 2025, 3^3^	subgen. nov.	*Borrelia burgdorferi*	11	[23]
*Brachybacterium timonense* Kuete *et al*. 2019, 3^3,20^	sp. nov.	Marseille-P4339 (=CECT 9821=CSUR P4339)	135	[24]
*Bradyrhizobium aeschynomenes* Sun *et al*. 2022, 10^3^	sp. nov.	83002 (=KCTC 82266=MCCC 1K04775)	43	[25]
*Buttiauxella massiliensis* Zgheib *et al*. 2022, 7^3^	sp. nov.	Marseille-P9829 (=CSUR P9829=DSM 110695)	141	[26]
*Caballeronia ginsengisoli* Quan *et al*. 2019, 447	sp. nov.	Gsoil 652 (=KACC 19441-LMG 30326)	27	[27]
*Chelativorans xinjiangensis* corrig. Meng *et al*. 2021, 697^21^	sp. nov.	lm93 (=CCTCC AB 2019376=KCTC 72857)	152	[28]
*Chryseobacterium hilariae* Kämpfer *et al*. 2025, 12^3^	sp. nov.	DT-3 (=CCM 9258=CIP 112171=LMG 32722)	95	[29]
*Chryseolinea lacunae* Kim *et al*. 2023, 5^3^	sp. nov.	Jin1 (=KCTC 82562=NBRC 114837)	2	[30]
*Clostridium caccae* corrig. Tall *et al*. 2020, 8^3,22^	sp. nov.	Marseille-P4344 (=CECT 9785=CSUR P4344)^23^	133	[31]
*Clostridium massiliamazoniense* Dione *et al*. 2020, 2012	sp. nov.	ND2 (=CECT 30352=CSUR P1360)^24^	137	[32]
*Cognatundibacterium* Lu *et al*. 2025, 9^3,25^	gen. nov.	*Cognatundibacterium oligocarboniphilum*	101	[6]
*Cognatundibacterium aquatile* (Du *et al*. 2015) Lu *et al*. 2025, 10^3^	comb. nov. [basonym: *Undibacterium aquatile* Du *et al*. 2015]	THG-DN7.3 (=CCTCC AB 2015119=KCTC 42243)	101	[6]
*Cognatundibacterium crateris* (Phurbu *et al*. 2021) Lu *et al*. 2025, 10^3^	comb. nov. [basonym: *Undibacterium crateris* Phurbu *et al*. 2021]	B2R-29 (=CGMCC 1.13792=KCTC 72018)	101	[6]
*Cognatundibacterium curvum* (Lu *et al*. 2021) Lu *et al*. 2025, 10^3^	comb. nov. [basonym: *Undibacterium curvum* Lu *et al*. 2021]	CY22W (=GDMCC 1.1906=KACC 21951)	101	[6]
*Cognatundibacterium griseum* (Lu *et al*. 2021) Lu *et al*. 2025, 10^3^	comb. nov. [basonym: *Undibacterium griseum* Lu *et al*. 2021]	FT31W (=GDMCC 1.1908=KACC 21953)	101	[6]
*Cognatundibacterium jejuense* (Kim *et al*. 2014) Lu *et al*. 2025, 10^3^	comb. nov. [basonym: *Undibacterium jejuense* Kim *et al*. 2014]	JS4-4 (=KACC 12607=NBRC 108922)^26^	101	[6]
*Cognatundibacterium luofuense* (Lu *et al*. 2021) Lu *et al*. 2025, 10^3^	comb. nov. [basonym: *Undibacterium luofuense* Lu *et al*. 2021]	LFS511W (=GDMCC 1.2458=KCTC 82658)	101	[6]
*Cognatundibacterium oligocarboniphilum* (Eder *et al*. 2011) Lu *et al*. 2025, 10^3^	comb. nov. [basonym: *Undibacterium oligocarboniphilum* Eder *et al*. 2011]	EM 1 (=CCUG 57265=DSM 21777)	101	[6]
*Cognatundibacterium rivi* (Lu *et al*. 2021) Lu *et al*. 2025, 10^3^	comb. nov. [basonym: *Undibacterium rivi* Lu *et al*. 2021]	FT147W (=GDMCC 1.2457=KCTC 82657)	101	[6]
*Cognatundibacterium rugosum* (Lu *et al*. 2021) Lu *et al*. 2025, 10^3^	comb. nov. [basonym: *Undibacterium rugosum* Lu *et al*. 2021]	CY7W (=GDMCC 1.1903=KACC 21961)	101	[6]
*Cognatundibacterium squillarum* (Sheu *et al*. 2014) Lu *et al*. 2025, 10^3^	comb. nov. [basonym: *Undibacterium squillarum* Sheu *et al*. 2014]	CMJ-15 (=BCRC 80404=KCTC 23917)^27^	101	[6]
*Cohnella abietis* Jiang *et al*. 2019, 957	sp. nov.	HS21 (=CCTCC AB 2019010=KCTC 43028)	87	[33]
*Coprococcus intestinalis* Shamsuzzaman *et al*. 2025, 11^3,28^	sp. nov.	B2-R-112 (=CGMCC 1.17967=KCTC 25419)	85	[34]
*Corynebacterium incognitum* Boxberger *et al*. 2021, 5^3,29^	sp. nov.	Marseille-Q3630 (=CECT 30218=CSUR-Q3630)	119	[35]
*Corynebacterium pacaense* Bellali *et al*. 2019, 7^3,30^	sp. nov.	Marseille-P2417 (=CSUR P2417=DSM 104935)	122	[36]
*Croceibacterium segetis* (Lee and Whang 2025) Lin *et al*. 2025, 12^3^	comb. nov. [basonym: *Altererythrobacter segetis* Lee and Whang 2025]	YJ20 (=KACC 19554=NBRC 113199)	130	[37]
*Cronobacter dublinensis* subsp. *beijingensis* Gan *et al*. 2025, 10^3^	subsp. nov.	CFSA2021A14 (=CGMCC 1.61450=JCM 35886)	51	[38]
*Cronobacter dublinensis* subsp. *infanticibi* Gan *et al*. 2025, 11^3^	subsp. nov.	CFSA2021A84 (=CGMCC 1.61451=JCM 35887)	51	[38]
*Cupriavidus malaysiensis* Ramachandran *et al*. 2018, 369	sp. nov.	USMAA1020 (=DSM 19416=KCTC 32388)	39	[39]
*Curtobacterium salicis* Freeman *et al*. 2025, 1^3^	sp. nov.	WW7 (=DSM 120164=NCCB 101076)	49	[40]
*Curvibacter cyanobacteriorum* Le *et al*. 2023, 1435	sp. nov.	HBC61 (=KCTC 92794=LMG 32713)	110	[41]
*Curvibacter microcysteis* Le *et al*. 2023, 1435	sp. nov.	RS43 (=KCTC 92793=LMG 32714)	110	[41]
*Deinococcus lichenicola* Chen *et al*. 2025, 7^3^	sp. nov.	YIM 134068 (=CGMCC 1.61729=MCCC 1K08693=NBRC 117087)	40	[42]
*Desulfoluna limicola* Watanabe *et al*. 2022, 4^3^	sp. nov.	ASN36 (=DSM 111985=JCM 39257)	139	[43]
*Devosia oryzisoli* Chhetri *et al*. 2023, 238	sp. nov.	PTR5 (=KCTC 82691=TBRC 15163)	35	[44]
*Draconibacterium halophilum* Kim *et al*. 2021, 2444	sp. nov.	M1 (=KCTC 72809=VTCC 910107)	106	[45]
*Duganella caerulea* Kishiro *et al*. 2025, 9^3^	sp. nov.	R57 (=DSM 115070=NBRC 115983)	48	[46]
*Duganella hordei* Kishiro *et al*. 2025, 9^3^	sp. nov.	R1 (=DSM 115069=NBRC 115982)	48	[46]
*Duganella rhizosphaerae* Kishiro *et al*. 2025, 12^3^	sp. nov.	R64 (=DSM 115071=NBRC 115984)	48	[46]
*Erwinia artemisiae* Kämpfer *et al*. 2025, 12^3^	sp. nov.	DT-104 (=CIP 112176=DSM 114512=LMG 32725)^31^	95	[29]
*Ferrovibrio plantarum* Kang *et al*. 2025, 6^3^	sp. nov.	MS7 (=KCTC 8000=LMG 33141)	97	[47]
*Flagellimonas alginilytica* corrig. Yu *et al*. 2025, 14^3,32^	sp. nov.	C4 (=KCTC 92864=MCCC M28979)	15	[48]
*Flagellimonas cixiensis* Yu *et al*. 2025, 14^3,33^	sp. nov.	GZD32 (=KCTC 102298=MCCC 1K09106)	15	[48]
*Flavobacterium jocheonense* corrig. Choi *et al*. 2019, 1268 ^34^	sp. nov.	UR11 (=JCM 31512=KCTC 52377)	111	[49]
*Flavobacterium nakdongense* corrig. Le *et al*. 2024, 2249 ^35^	sp. nov.	20NA77.7 (=KCTC 102000=LMG 33137)	112	[50]
*Flavobacterium panacisoli* Jung *et al*. 2016, 649^36^	sp. nov.	DCY70 (=JCM 19162=KCTC 32393)	145	[51]
*Fulverythrobacter* Lin *et al*. 2025, 11^3^	gen. nov.	*Fulverythrobacter ferritrahens*	118	[37]
*Fulverythrobacter ferritrahens* Lin *et al*. 2025, 11^3^	sp. nov.	CC-YST694 (=BCRC 81283=JCM 34166)	118	[37]
*Fulverythrobacter fulvus* (Dahal and Kim 2018) Lin *et al*. 2025, 11^3^	comb. nov. [basonym: *Altererythrobacter fulvus* Dahal and Kim 2018]	S-54 (=KACC 19119=NBRC 112676)^37^	118	[37]
*Furfurilactobacillus entadae* Suzuki *et al*. 2025, 5^3^	sp. nov.	OKN36 (=DSM 118293=JCM 37107)	44	[52]
*Fusobacterium paranimalis* Sivertsen *et al*. 2025, 7^3^	sp. nov.	Vestland19 (=DSM 119817=NCTC 15132)	73	[53]
*Govanella* Vandamme *et al*. 2025, 1^3^	gen. nov.	*Govanella unica*	63	[54]
*Govanella unica* Vandamme *et al*. 2025, 1^3^	sp. nov.	LMG 31809 (=CECT 30155)	63	[54]
*Govanellaceae* Vandamme *et al*. 2025, 1^3^	fam. nov.	*Govanella*	63	[54]
*Gracilimonas qinghaiensis* Wang *et al*. 2025, 14^3^	sp. nov.	Q87 (=KCTC 102199=MCCC 1K09482)	13	[55]
*Granulicatella seriolae* Lee *et al*. 2024, 6^3^	sp. nov.	S8 (=JCM 35604=KCTC 43438)	57	[56]
*Haloimpatiens myeolchijeotgali* Lee and Chun 2025, 7^3^	sp. nov.	FM7330 (=JCM 37575=KCTC 25938)	81	[57]
*Haloimpatiens sporogenes* Lee and Chun 2025, 7^3^	sp. nov.	FM7315 (=JCM 37574=KCTC 25939)	81	[57]
*Halopseudomonas nanhaiensis* (Pang *et al*. 2021) Girard *et al*. 2023, 16^3,38^	comb. nov. [basonym: *Pseudomonas nanhaiensis* Pang *et al*. 2021]	SCS 2-3 (=GDMCC 1.2219=JCM 34440)	10	[58]
*Helicobacter higonensis* Tomida *et al*. 2024, 203^39^	sp. nov.	PAGU2000 (=GTC 16811=LMG 33095)	64	[59]
*Histidinibacterium aquaticum* Lee and Park 2022, 5^3^	sp. nov.	176SS1-4 (=KACC 19891=LMG 31032)	23	[60]
*Hymenobacter aranciens* Kim *et al*. 2024, 8^3,40^	sp. nov.	ASUV-10-1 (=KCTC 92969=NBRC 116575)	92	[61]
*Hymenobacter pini* Bang *et al*. 2022, 6^3^	sp. nov.	BT728 (=KACC 22629=NBRC 115444)	90	[62]
*Hymenobacter ruricola* Bang *et al*. 2021, 1137	sp. nov.	BT662 (=KACC 21966=NBRC 114855)	69	[63]
*Kingella bonacorsii* Antezack *et al*. 2021, 11^3,41^	sp. nov.	Marseille-Q4569 (=CECT 30237=CSUR Q4569)	120	[64]
*Kueselia* Kündgen *et al*. 2025, 9^3^	gen. nov.	*Kueselia aquadivae*	58	[65]
*Kueselia aquadivae* Kündgen *et al*. 2025, 11^3^	sp. nov.	EP7 (=CECT 30426=STH00993)	58	[65]
*Labilibaculum euxinicum* corrig. Yadav *et al*. 2020, 8^3,42^	sp. nov.	A4 (=JCM 32481=KCTC 15662)	22	[66]
*Larkinella bombi* (Zhang *et al*. 2024) Quadri *et al*. 2025, 416	comb. nov. [basonym: *Tellurirhabdus bombi* Zhang *et al*. 2024]	IE-0392 (=GDMCC 1.2794=JCM 35040)	66	[67]
*Larkinella roseola* Quadri *et al*. 2025, 416	nom. nov. [basonym: *Tellurirhabdus rosea* Choi *et al*. 2021]	U15 (=JCM 32361=KCTC 62116)	66	[67]
*Leucobacter manosquensis* Boxberger *et al*. 2023, 10^3^	sp. nov.	Marseille-Q4368 (=CSUR Q4368=DSM 112403)	134	[68]
*Luteimonas dalianensis* Xin *et al*. 2014, 732^43^	sp. nov.	OB44-3 (=CGMCC 1.12191=JCM 18136)	1	[69]
*Lysobacter tongrenensis* Li *et al*. 2018, 442	sp. nov.	YS-37 (=CCTCC AB 2016052=KCTC 52206)	145	[70]
*Marinobacter gelidimuriae* Chua *et al*. 2018, 11^3^	sp. nov.	BF04_CF4 (=ATCC TSD-107=DSM 106148)^44^	6	[71]
*Marinobacterium arenosum* Lee *et al*. 2022, 4^3^	sp. nov.	CAU 1594 (=KCTC 82405=MCCC 1K05672)	143	[72]
*Massilia phyllosphaerae* Yao *et al*. 2025, 10^3^	sp. nov.	SGZ-792 (=GDMCC 1.4211=JCM 36643)	151	[73]
*Massilia pseudoviolaceinigra* Sedláček *et al*. 2025, 9^3^	sp. nov.	P3689 (=CCM 9206=LMG 33568)	80	[74]
*Massilia scottii* Sedláček *et al*. 2025, 8^3^	sp. nov.	P5043 (=CCM 9029=LMG 32502)	80	[74]
*Microbacterium deferens* corrig. Lustermans *et al*. 2025, 8^3,45^	sp. nov.	A1-JK (=DSM 119944=LMG 34034)	14	[75]
*Microbacterium rhizophilum* corrig. Liu *et al*. 2025, 8^3,46^	sp. nov.	GXG1230 (=KCTC 59252=MCCC 1K09302)	65	[76]
*Microbulbifer hainanensis* Cheng *et al*. 2021, 1038 ^37^	sp. nov.	NBU-8HK146 (=KCTC 82226=MCCC 1K04737)	3	[77]
*Microcella aerolata* Pan *et al*. 2022, 5^3^	sp. nov.	GA224 (=GDMCC 1.2165=JCM 34462)	70	[78]
*Micromonospora reichwaldensis* Nouioui *et al*. 2025, 12^3^	sp. nov.	Asg4 (=DSM 115977=KCTC 59188)	100	[79]
*Microvirga splendida* Park *et al*. 2022, 746^47^	sp. nov.	BT325 (=KCTC 72406=NBRC 114847)	36	[80]
*Moraxella marmotae* Liu *et al*. 2025, 7^3^	sp. nov.	ZJ142 (=GDMCC 1.3113=JCM 35324)	38	[81]
*Mycobacterium antarcticum* Shimada *et al*. 2025, 10^3^	sp. nov.	TUM20985 (=NCTC 15058=RIMD 4000203)	123	[82]
*Negativicoccus massiliensis* Togo *et al*. 2020, 1007	sp. nov.	Marseille-P2082 (=CSUR P2082=DSM 100853)	147	[83]
*Neiella holothuriorum* Bai *et al*. 2022, 502	sp. nov.	126 (=GDMCC 1.2530=KCTC 82829)	5	[84]
*Neobacillus massiliamazoniensis* Mbaye *et al*. 2021, 6^3,48^	sp. nov.	LF1 (=CECT 30364=CSUR P1359)	121	[85]
*Neorhizobium descurainiae* Kämpfer *et al*. 2025, 12^3^	sp. nov.	DT-125 (=CIP 112184=LMG 32760)	95	[29]
*Neoundibacterium* Ma *et al*. 2023, 8^3^	gen. nov.	*Neoundibacterium parvum*	99	[5]
*Neoundibacterium parvum* (Eder *et al*. 2011) Ma *et al*. 2023, 8^3^	comb. nov. [basonym: *Undibacterium parvum* Eder *et al*. 2011]	CIP 109317 (=CCUG 49012=DSM 23061)	99	[5]
*Neoundibacterium piscinae* (Lee *et al*. 2019) Ma *et al*. 2023, 8^3^	comb. nov. [basonym: *Undibacterium piscinae* Lee *et al*. 2019]	S11R28 (=JCM 33224=KCTC 62668)	99	[5]
*Nibribacter flagellatus* Lin *et al*. 2018, 1783	sp. nov.	THG-T16 (=CCTCC AB 2016246=KACC 19188)	84	[86]
*Paenibacillus albilobatus* Lee *et al*. 2018, 397	sp. nov.	h2 (=JCM 32395=LMG 30408)^49^	75	[87]
*Paenibacillus ginsengiterrae* Huq *et al*. 2015, 394^50^	sp. nov.	DCY89 (=JCM 19887=KCTC 33430)	103	[88]
*Paenibacillus insulae* Cho *et al*. 2015, 590	sp. nov.	DS80 (=JCM 17278=KCTC 13833)	46	[89]
*Paenibacillus silvisoli* Lee *et al*. 2024, 4^3^	sp. nov.	TW38 (=KCTC 43468=NBRC 116015)	107	[90]
*Paenibacillus tumbae* Huang *et al*. 2017, 362	sp. nov.	CSA42 (=CCTCC AB 2016201=KACC 18941)	45	[91]
*Pampinifervens* Palmer *et al*. 2025, 8^3^	gen. nov.	*Pampinifervens diazotrophicum*	76	[92]
*Pampinifervens diazotrophicum* Palmer *et al*. 2025, 8^3^	sp. nov.	T-2 (=DSM 116324=JCM 35475)	76	[92]
*Pampinifervens florentissimum* Palmer *et al*. 2025, 8^3^	sp. nov.	T-8 (=CGMCC 1.5214=JCM 33569)^51^	76	[92]
*Parabacteroides absconsus* Chaplin *et al*. 2025, 11^3,52^	sp. nov.	AD58 (=JCM 35468=VKM B-3630)	42	[93]
*Parabacteroides caeci* Shamsuzzaman *et al*. 2025, 14^3^	sp. nov.	W1-Q-101 (=CGMCC 1.17991=KCTC 25456)	31	[94]
*Parabacteroides caecihominis* Shamsuzzaman *et al*. 2025, 12^3^	sp. nov.	TA-V-105 (=CGMCC 1.17989=KCTC 25451)	85	[34]
*Paracoccus aurantius* Hwang *et al*. 2024, 2021	sp. nov.	MBLB3053 (=JCM 36634=KCTC 8269)^53^	113	[95]
*Paracoccus maritimus* Yu *et al*. 2024, 5^3^	sp. nov.	YLB-12 (=KCTC 82197=MCCC 1A17213)	4	[96]
*Parapedobacter brassicae* Kämpfer *et al*. 2025, 12^3^	sp. nov.	DT-150 (=DSM 115120=LMG 32759)	95	[29]
*Parasphingorhabdus halotolerans* Kim *et al*. 2021, 3805^54^	sp. nov.	JK6 (=KCTC 72818=VTCC 910111)	104	[97]
*Parundibacterium* corrig. Ma *et al*. 2023, 8^3,55^	gen. nov.	*Parundibacterium terreum* corrig.	99	[5]
*Parundibacterium terreum* corrig. (Liu *et al*. 2013) Ma *et al*. 2023, 9^3^	comb. nov. [basonym: *Undibacterium terreum* Liu *et al*. 2013]	C3 (=CGMCC 1.10998=NBRC 108789)	99	[5]
*Pelagibacterium mangrovi* Yang *et al*. 2025, 7^3^	sp. nov.	WS-203 (=GDMCC 1.4694=KCTC 8652)	17	[98]
*Peptoniphilus mikwangii* Cho *et al*. 2015, 265	sp. nov.	CHdc B134 (=JCM 30223=KCTC 15227)^56^	30	[99]
*Planococcus dechangensis* Wang *et al*. 2015, 1081	sp. nov.	NEAU-ST10-9 (=CGMCC 1.12151=DSM 25871)	7	[100]
*Polaribacter gochangensis* Kathiresan *et al*. 2025, 11^3^	sp. nov.	MF5-112 (=JCM 37723=KCTC 102318=KEMB 23950)	20	[101]
*Polaribacter sargassicola* Li *et al*. 2025, 9^3^	sp. nov.	DS7-9 (=KCTC 102076=MCCC 1K08670)	18	[9]
*Prevotella marseillensis* Kuete Yimagou *et al*. 2019, 2^3,57^	sp. nov.	Marseille-P8229 (=CECT 30037=CSUR P8229)^22^	136	[102]
*Providencia lanzhouensis* Xiang *et al*. 2025, 12^3^	sp. nov.	PAZ2 (=CCTCC AB 2024284=KCTC 8881)	83	[103]
*Pseudomonas bambusae* He *et al*. 2025, 9^3^	sp. nov.	RC10 (=MCCC 1K08846=JCM 36624)	37	[104]
*Pseudomonas chlororaphis* subsp. *phenazini* Chi *et al*. 2024, 11^3^	subsp. nov.	S1Bt23 (=CFBP 9180=LMG 33497)	141	[105]
*Pseudomonas nanhaiensis* Pang *et al*. 2021, 1801^58^	sp. nov.	SCS 2–3 (=GDMCC 1.2219=JCM 34440)	9	[106]
*Pseudomonas parasichuanensis* Tohya *et al*. 2023, 2^3^	sp. nov.	BML-PP020 (=JCM 34580=LMG 32249)	29	[107]
*Pseudomonas tumuqii* Kong *et al*. 2022, 5^3^	sp. nov.	LAMW06 (=GDMCC 1.2003=KCTC 72829)	8	[108]
*Pseudomonas wuhanensis* Hou *et al*. 2024, 9^3,59^	sp. nov.	FP607 (=ACCC 62446=JCM 35688)^60^	74	[109]
*Pseudonocardia phyllosphaerae* Khanoonkon *et al*. 2025, 8^3^	sp. nov.	1LY6.1 (=NBRC 116933=TBRC 19001)	125	[110]
*Pseudoxanthomonas composti* Lin *et al*. 2019, 1217	sp. nov.	GSS15 (=KCTC 52974=MCCC 1K03334)	102	[111]
*Pseudoxanthomonas humi* Akter *et al*. 2015, 1170^61^	sp. nov.	THG-MM13 (=CCTCC AB 2015122=KACC 18280)	32	[112]
*Pseudundibacterium* Lu *et al*. 2025, 9^3,62^	gen. nov.	*Pseudundibacterium arcticum*	101	[6]
*Pseudundibacterium arcticum* (Li *et al*. 2016) Lu *et al*. 2025, 11^3^	comb. nov. [basonym: *Undibacterium arcticum* Li *et al*. 2016]	6-67 (=CCTCC AB 2015162=KCTC 42986)	101	[6]
*Psychrilyobacter piezotolerans* Yadav *et al*. 2021, 907	sp. nov.	SD5 (=JCM 32482=KCTC 15663)	60	[113]
*Qipengyuania algicola* Lee *et al*. 2025, 9^3^	sp. nov.	DGS2-2 (=JCM 37496=KACC 23855)	114	[114]
*Qipengyuania rhodophyticola* Lee *et al*. 2025, 10^3^	sp. nov.	DGS5-3 (=JCM 37497=KACC 23854)	114	[114]
*Raoultibacter phocaeensis* Yacouba *et al*. 2022, 7^3,63^	sp. nov.	Marseille-P8396 (=CECT 30202=CSUR P-8396)	148	[115]
*Rhodohalobacter sulfatireducens* Wang *et al*. 2022, 8^3^	sp. nov.	WB101 (=KCTC 92204=MCCC 1H00518)	33	[116]
*Roseateles agri* You and Kim 2024, 9^3^	sp. nov.	R3-3 (=KACC 23678=NBRC 116681)	149	[117]
*Roseobacter weihaiensis* Li *et al*. 2025, 5^3,39^	sp. nov.	H9 (=KCTC 82507=MCCC 1K04354)	86	[118]
*Roseomonas chloroacetimidivorans* Chu *et al*. 2016, 610	sp. nov.	BUT-13 (=CCTCC AB 2015299=JCM 31050)	129	[119]
*Roseomonas rosulenta* Lee and Whang 2022, 4^3^	sp. nov.	OP-27 (=KACC 21501=NBRC 114497)	53	[120]
*Rufibacter radiotolerans* Kim and Srinivasan 2021, 352	sp. nov.	DG31D (=JCM 19446=KCTC 32454)	71	[121]
*Rugositalea* Chung *et al*. 2025, 16^3^	gen. nov.	*Rugositalea oryzae*	50	[122]
*Rugositalea oryzae* Chung *et al*. 2025, 16^3,64^	sp. nov.	YC6860 (=KCCM 43037=NBRC 109316)	50	[122]
*Salinimonas marina* Kim *et al*. 2021, 3325	sp. nov.	G2-b (=KCTC 72817=VTCC 910110)^65^	52	[123]
*Schaalia dentiphila* Tian *et al*. 2024, 15^3^	sp. nov.	NCTC 9931 (=CCUG 18309=CIP 104728=DSM 43331)^66^	94	[124]
*Segatella intestinalis* Shamsuzzaman *et al*. 2025, 13^3^	sp. nov.	B2-R-102 (=CGMCC 1.17963=KCTC 25417)	31	[94]
*Selenomonas felix* Kuete *et al*. 2019, 3^3,67^	sp. nov.	Marseille-P3560 (=CECT 9822=CSUR P3560)	132	[125]
*Shewanella psychropiezotolerans* Yu *et al*. 2021, 6^3,68^	sp. nov.	YLB-06 (=KCTC 62907=MCCC 1A12715)	89	[126]
*Sinimarinibacterium arenosum* Kim *et al*. 2020, 4^3^	sp. nov.	CAU 1509 (=KCTC 72000=NBRC 113698)	47	[127]
*Sinomonas terrae* Lee *et al*. 2023, 913	sp. nov.	5–5 (=KCTC 49650=NBRC 115790)	115	[128]
*Siphonobacter intestinalis* Lee *et al*. 2016, 711	sp. nov.	63MJ-2 (=KACC 18663=NBRC 111883)	56	[129]
*Sneathiella litorea* Khan *et al*. 2021, 810	sp. nov.	DP05 (=JCM 33810=KACC 21431)	68	[130]
*Sphingomonas abietis* Jiang *et al*. 2023, 1297	sp. nov.	PAMB 00755 (=GDMCC 1.3779=KCTC 92781)	116	[131]
*Sphingomonas astragali* Kämpfer *et al*. 2025, 15^3^	sp. nov.	DT-204 (=CCM 9255=CIP 112175=LMG 33194)	95	[29]
*Sphingomonas immobilis* Choi *et al*. 2024, 9^3,69^	sp. nov.	CA1-15 (=KCTC 92960=NBRC 116547)	79	[132]
*Sphingomonas larreae* Kämpfer *et al*. 2025, 15^3^	sp. nov.	DT-51 (=CCM 9259=CIP 112177=DSM 114511=LMG 32723)	95	[29]
*Sphingomonas radicis* Kämpfer *et al*. 2025, 15^3^	sp. nov.	DT-207 (=CCM 9257=DSM 114514=LMG 32727)	95	[29]
*Sphingopyxis microcysteis* Zhang *et al*. 2021, 853	sp. nov.	Z10-6 (=CCTCC AB2017276=KCTC 62492)	55	[133]
*Staphylococcus halotolerans* Baek *et al*. 2025, 7^3^	sp. nov.	KG4-3 (=JCM 37037=KACC 23684)	41	[134]
*Streptococcus atopicus* corrig. Chan *et al*. 2025, 6^3,70^	sp. nov.	AD045-1 (=BCRC 81461=NBRC 117137)	146	[135]
*Streptomyces albidicamelliae* corrig. Long *et al*. 2024, 790^71^	sp. nov.	HUAS 14-6 (=JCM 35920=MCCC 1K08365)	28	[136]
*Streptomyces chengbuensis* Zheng *et al*. 2024, 574^72^	sp. nov.	HUAS CB01 (=JCM 36277=MCCC 1K08666)^73^	124	[137]
*Streptomyces cynarae* Deng *et al*. 2023, 1281	sp. nov.	HUAS 13-4 (=JCM 35919=MCCC 1K08364)^73^	128	[138]
*Streptomyces humi* Zainal *et al*. 2016, 472	sp. nov.	MUSC 119 (=DSM 42174=MCCC 1K00505)	96	[139]
*Streptomyces lacaronensis* Calvo-Peña *et al*. 2025, 17^3,74^	sp. nov.	OE57 (=CECT 31164=DSM 118741)	98	[140]
*Streptomyces secundicynarae* Song *et al*. 2025, 598	sp. nov.	HUAS ZL42 (=JCM 37325=MCCC 1K09444)	131	[141]
*Streptomyces tabacisoli* Li *et al*. 2025, 233	sp. nov.	HUAS MG91 (=JCM 37027=MCCC 1K09288)	138	[142]
*Streptomyces triticiradicis* Yu *et al*. 2020, 10^3^	sp. nov.	NEAU-H2 (=CCTCC AA 2018031=DSM 109825)	150	[143]
*Sulfitobacter albidus* Kim *et al*. 2022, 6^3^	sp. nov.	JK7-1 (=KCTC 72819=NBRC 114632)^75^	153	[144]
*Sulfitobacter aquimarinus* Shin *et al*. 2024, 250	sp. nov.	HNIBRBA2951 (=KCTC 8586=MCCC 1K09399)	72	[145]
*Sulfitobacter marinivivus* Shin *et al*. 2024, 250	sp. nov.	HNIBRBA3233 (=KCTC 8585=MCCC 1K09401)	72	[145]
*Sulfitobacter rhodophyticola* Choi *et al*. 2025, 9^3^	sp. nov.	JB4-11 (=JCM 36646=KACC 23867)	77	[146]
*Sulfitobacter sediminivivens* Kathiresan *et al*. 2025, 10^3^	sp. nov.	MF3-043 (=KCTC 8706=TBRC 19386)	19	[147]
*Thermoanaerobacter ethanolicus* subsp. *ethanolicus* (Wiegel and Ljungdahl 1981) Bing 2024, 175	comb. nov. [basonym: *Thermoanaerobacter ethanolicus* Wiegel and Ljungdahl 1981]	JW 200 (=ATCC 31550=DSM 2246)	54	[148]
*Thermoanaerobacter ethanolicus* subsp. *indiensis* Bing 2024, 175	subsp. nov.^76^	BSB-33 (=ATCC BAA-2171=DSM 25103)	54	[148]
*Thermoanaerobacter ethanolicus* subsp. *thermohydrosulfuricus* (Klaushofer and Parkkinen 1965) Bing 2024, 176	comb. nov. [basonym: *Clostridium thermohydrosulfuricum* Klaushofer and Parkkinen 1965 (Approved Lists 1980)]	E100-69 (=ATCC 3504=CECT 594=DSM 567=DSM 25103=NCIMB 10956)^77^	54	[148]
*Variovorax stachyos* Kämpfer *et al*. 2025, 12^3^	sp. nov.	DT-64 (=CIP 112170=DSM 114531=LMG 32724)	95	[29]
*Variovorax terrae* Woo and Kim 2022, 859	sp. nov.	CYS-02 (=KACC 22656=NBRC 115645)^78^	117	[149,150]
*Variovorax xiamenensis* Wang *et al*. 2025, 9^3,79^	sp. nov.	W6 (=KCTC 8209=MCCC 1K08691)	12	[151]
*Vibrio aquimaris* Franco *et al*. 2020, 14^3^	sp. nov.	THAF100 (=CIP 111709=DSM 109633=LMG 31434)	24	[152]
*Vibrio profundi* Zhang *et al*. 2019, 1606	sp. nov.	TP187 (=CGMCC 1.15395=KACC 18555)	26	[153]

For references to Validation Lists 1–71, see *Int J Syst Bacteriol* **49** (1999) 1325. Lists 72–197 were published in *Int J Syst Evol Microbiol* **50** (2000) 3, 423, 949, 1415, 1699, 1953; and **51** (2001) 1, 263, 793, 1229, 1619, 1945; and **52** (2002) 3, 685, 1075, 1437, 1915; and **53** (2003) 1, 373, 627, 935, 1219, 1701; and **54** (2004) 1, 307, 631, 1005, 1425, 1909; and **55** (2005) 1, 547, 983, 1395, 1743, 2235; and **56** (2006) 1, 499, 925, 1459, 2025, 2507; and **57** (2007) 1, 433, 893, 1371, 1933, 2449; and **58** (2008) 1, 529, 1057, 1511, 1993, 2471; and **59** (2009) 1, 451, 923, 1555, 2129, 2647; and **60** (2010) 1, 469, 1009, 1477, 1985, 2509; **61** (2011) 1, 475, 1011, 1499, 2025, 2563; and **62** (2012) 1, 473, 1017, 1443, 2045, 2549; and **63** (2013) 1, 797, 1577, 2365, 3131, 3931; and **64** (2014) 1, 693, 1455, 2184, 3603; and **65** (2015), 1, 741, 1112, 2017, 2777, 3767; and **66** (2016) 1, 1603, 1913, 2463, 3761, 4299; and **67** (2017) 1, 529, 1095, 2075, 3140, 4291; and **68** (2018), 1, 693, 1411, 2130, 2707, 3379; and **69** (2019), 5, 597, 1247, 1844, 2627, 3313; and **70 **(2020) 1, 1443, 2960, 4043, 4844, 5596. Lists 197-226 were published in *Int J Syst Evol Microbiol* **71** (2021) 004600, 004688, 004773, 004846, 004943, 005096; and **72** (2022) 005167, 005260, 005331, 005422, 005517, 005592; and **73** (2023) 005709, 005812, 005845, 005931, 005997, 006080; and **74 **(2024) 006173, 006229, 006275, 006398, 006452, 006501; and **75 **(2025) 006562, 006640, 006699, 006863, 006910.

1Abbreviations of culture collections cited in this list can be found at https://lpsn.dsmz.de/text/collections.

2Priority number assigned according to the date the documentation and request for validation are received.

3The journal in which the name was effectively published does not have continuous pages.

4The list editors have corrected *Actinospongicola *to *Actinospongiicola *(Ac.ti.no.spon.gi.i’co.la. Gr. fem. n. *aktis*, ray, beam; L. fem. n. *spongia*, sponge; L. suff. -*cola *(from L. masc./fem. n. *incola*), inhabitant, dweller; N.L. masc. n. *Actinospongiicola,* a member of the *Actinomycetota *inhabiting sponges]. The authors did not assign a gender to the genus name; the list editors propose masculine.

5The authors did not explicitly state the type species, but the genus includes only one species.

6The effective publication states that the type strain was also deposited as CGMCC 1.15063 and KCTC 39113, but no documentation was supplied.

7The effective publication states that the type strain was also deposited as LMG 29730, but no documentation was supplied.

8The effective publication states that the type strain was also deposited as DSM 102221, but no documentation was supplied.

9The authors gave Shen instead of Sheu.

10The effective publication states that the type strain was also deposited as BCRC 80406 and LMG 26891, but no documentation was supplied.

11The list editors have corrected *deltaense *to *deltae* (del’tae. L. gen. n. *deltae*, of a river delta).

12The protologue heading must state *Algoriphagus limi *sp. nov. instead of *A. limi *sp. nov.

13Syllabification and etymology must be given as se.ne.gal.en’sis. N.L. masc. adj. *senegalensis*.

14The protologue heading must state *aquimaris* instead of *Aquimaris*.

15Etymology must be given as follows: L. fem. n. *vagina*, sheath, vagina; L. fem. n. *Massilia*, the Latin name of Marseille; N.L. masc. adj. *vaginimassiliensis*, referring to the vagina and to Marseille where the type strain was isolated.

16No culture collection accession numbers are mentioned in the protologue; one number is mentioned in the abstract.

17The list editors have corrected *formicoacetica* to *formicacetica* as the basonym was corrected to *Clostridium formicaceticum*.

18Syllabification and etymology must be given as poe.cil.las.tri’co.la. N.L. fem. n. *Poecillastra*, a sponge genus; N.L. masc. n. *poecillastricola*.

19The preferred etymology is L. masc. adj. *brevis*.

20Etymology must state N.L. neut. adj. instead of N.L. masc. adj.

21The list editors have corrected *xinjiangense* to *xinjiangensis* (xin.jiang.en’sis. N.L. masc. adj. *xinjiangensis*).

22The list editors have corrected the epithet to *caccae* (cac’cae. Gr. fem. n. *kakke*, human ordure, faeces; N.L. gen. n. *caccae*, of faeces).

23The CSUR number is mentioned in the abstract, not in the protologue.

24The effective publication states that the type strain was also deposited as DSM 27309, but no documentation was supplied.

25The protologue heading must be gen. nov. instead of gen. Nov., and etymology L. masc. adj. instead of L. masc. Adj.

26The effective publication states that the type strain was also deposited as DSM 19386, but no documentation was supplied.

27The effective publication states that the type strain was also deposited as LMG 26892, but no documentation was supplied.

28The etymology must state N.L. masc. adj. instead of N.L. fem. adj. The authors gave an acknowledgement to Aharon Oren, but he did not suggest N.L. masc. adj.

29In the title, the epithet is misspelled *incognita*. The preferred etymology is: in.cog’ni.tum. L. neut. adj. *incognitum*, unknown.

30The etymology must be corrected as follows: N.L. neut. adj. *pacaense*, referring to ‘PACA’, the name of the region Provence-Alpes-Côte d’Azur, France.

31The effective publication states that the type strain was also deposited as CCM 9256, but no documentation was supplied.

32The list editors have corrected *alginolytica* to *alginilytica* (al.gi.ni.ly’ti.ca. N.L. fem. adj. *alginilytica*).

33Etymology must state N.L. fem. adj. instead of N.L. masc./fem. adj.

34The list editors have corrected *jocheonensis *to *jocheonense *(jo.che.on.en’sis. N.L. neut. adj. *jocheonense*).

35The list editors have corrected *nakdongensis *to *nakdongense *(nak.dong.en’sis. N.L. neut. adj. *nakdongensis*).

36Etymology must state pa.na.ci.so’li. N.L. masc. n. *Panax*, -*acis*, scientific name of ginseng; L. neut. n. *solum*, soil; N.L. gen. n. *panacisoli.*

37The effective publication states that the type strain was also deposited as KEMB 9005-542, but no documentation was supplied.

38Etymology must be corrected to N.L. fem. adj. *nanhaiensis.*

39Etymology must state N.L. masc. adj. instead of N.L. fem. adj.

40The preferred etymology is: a.ran’ci.ens. Italian fem. n. *arancia*, orange; N.L. masc. part. adj. *aranciens*, becoming orange-coloured.

41The preferred syllabification is: bo.na.cor’si.i.

42The list editors have corrected the epithet *euxinus* to *euxinicum* (eu.xi’ni.cum. N.L. neut. adj. *euxinicum*, pertaining to *Pontus euxinus* [hospitable], the Black Sea).

43Etymology must state N.L. fem. adj. instead of N.L. masc. adj.

44DSM 106148 instead of DSMZ 106148; the DSMZ document gives strain designation BF05_4.

45The list editors have corrected the epithet from *deferre* to *deferens* (de.fe’rens. L. neut. part. adj. *deferens*, transferring, transporting, named after the bacterium’s ability to perform electron transport via extracellular electron transfer). The protologue heading must give *Microbacterium *instead of *M*. The authors’ note ‘The species name *M. deferre *is proposed in accordance with the rules of the International Code of Nomenclature of Prokaryotes’ is incorrect.

46The list editors have corrected *rhizophilus *to *rhizophilum *(… N.L. neut. adj. *rhizophilum*, root-loving).

47The etymology must be completed as follows: … L. fem. adj. *splendida*, splendid.

48Syllabification and etymology must be given as mas.si.li.a.ma.zo.ni.en’sis. N.L. masc. adj. *massiliamazoniensis*.

49The effective publication states that the type strain was also deposited as KCCM 43269, but no documentation was supplied.

50Syllabification and etymology must be given as gin.sen.gi.ter’rae. N.L. neut. n. *ginsengum*, ginseng; L. fem. n. fem. n. *terra*, soil; N.L. gen. n. *ginsengiterrae*.

51The protologue table has CGMC instead of CGMCC.

52The preferred syllabification is abs.con’sus.

53In the abstract, MBLB3035 is given as MBL3035.

54The preferred etymology is L. pres. part. *tolerans*, tolerating.

55The list editors have corrected *Paraundibacterium *to *Parundibacterium*.

56The effective publication states that the type strain was also deposited as KCOM 1628, but no documentation was supplied.

57Syllabification and etymology must be given as mar.seil.len’sis. N.L. fem. adj. *marseillensis*, pertaining to Marseille.

58The epithet must be given as *nanhaiensis* instead of *Nanhaiensis*; syllabification nan.hai.en’sis. instead of Nan.hai.en’sis.

59The protologue heading must state *Pseudomonas* instead of* P.*

60The protologue gives ACCC 62,446 and JCM 35,688; the numbers in the abstract are correct. Also deposited as CGMCC 27,743 (CGMCC 27743), but no documentation was supplied.

61The protologue heading must state *Pseudoxanthomonas *instead of *P*.

62The protologue heading must state gen. nov. instead of gen. Nov. and etymology Gr. masc. adj. instead of Gr. masc. Adj.

63The etymology must state N.L. masc. adj. instead of N.L. adj. fem.

64The preferred syllabification is o.ry’zae.

65In the abstract, the strain number is given as G2-B.

66The effective publication states that the type strain was also deposited as ATCC 17982, CGMCC 1.90328 and NCTC 14978, but no documentation was supplied.

67The preferred etymology is L. fem. adj.

68The preferred syllabification psy.chro.pie.zo.to’le.rans.

69The preferred etymology is L. fem. adj. instead of L. masc./fem. adj.

70The list editors have corrected *atopica *to *atopicus *(a.to’pi.cus. N.L. masc. adj. *atopicus*, pertaining to atopy).

71The list editors have corrected *albidocamelliae *to *albidicamelliae*. The authors gave an acknowledgement to Aharon Oren, but he did not suggest *albidocamelliae*.

72The etymology must state N.L. masc. adj. instead of N.L. adj. fem.

73Culture collection numbers are given in the abstract, but not in the protologue.

74The etymology must state N.L. masc. adj. instead of N.L. gen. *N.*

75The effective publication states that the type strain was also deposited as KMM 6821, but no documentation was supplied.

76This is the effectively but not validly published *Thermoanaerobacter indiensis *Pal 2014.

77The effective publication states that the type strain was also deposited as LMG 6659, but no documentation was supplied.

78Originally given as NBRC 00115645, corrected in a corrigendum [151].

79The etymology must state N.L. masc. adj. instead of N.L. masc. Adj.

## Corrigenda

In Validation List no. 224 [154], the type strain *Pseudonocardia charpentierae* was deposited as DSM 45834 and not as DSM 45835 as listed.

In Validation List no. 225 [155], the following errors must be corrected:

*Anaerocellum diazotrophicum* corrig. (Chen et al. 2021) Bing 2024 cannot be considered validly published as the names of the genus *Anaerocellum* and its type species *Anaerocellum besci* were not validly published.

*Chryseosolibacter indicus* Octaviana *et al*. 2025 was correct in the effective publication and should not have been corrected by the List Editors to *Chryseosolibacter indicum*.

The DSM number for *Desulfurivibrio dismutans* AMeS2 should be DSM 113785 and not DSM 113758.

The DSM number for *Natronomicrosphaera hydrolytica* AB-hyl4 should be DSM 117794 and not DSM 117994.

We thank S. Spring (Leibniz Institute DSMZ, Braunschweig) and S. Ventura (IRET-CNR, Sesto Fiorentino and NBFC, Palermo, Italy) for alerting us to errors in Validation List no. 225.

## Abbreviations

ICNP: International Code of Nomenclature of Prokaryotes
*IJSEM*: *International Journal of Systematic and Evolutionary Microbiology*
